# The Association between Helicobacter pylori Infection and Glycated Hemoglobin A in Diabetes: A Meta-Analysis

**DOI:** 10.1155/2019/3705264

**Published:** 2019-09-09

**Authors:** Jinhu Chen, Yuling Xing, Liying Zhao, Huijuan Ma

**Affiliations:** ^1^Department of Endocrinology, Hebei General Hospital, Shijiazhuang 050017, China; ^2^Graduate School of Hebei Medical University, Shijiazhuang 050017, China; ^3^Hebei Key Laboratory of Metabolic Diseases, Hebei General Hospital Shijiazhuang, Hebei 050051, China; ^4^Department of Internal Medicine, Hebei Medical University, Shijiazhuang, Hebei 050017, China

## Abstract

**Background:**

The association between Helicobacter pylori infection and glycated hemoglobin A has been confirmed in many studies, but these conclusions are still contradictory and controversial. Therefore, we conducted a meta-analysis to resolve the problem of inconsistent results in diabetes.

**Methods:**

A comprehensive search was conducted on related researches published in PubMed, Embase, and China Academic Journal Full-text Database (CNKI) from the inception of each database to April 2019. Fixed or random effects model was used to pool the weighted mean difference with 95% confidence interval from individual studies. Subgroup and sensitivity analyses were also performed. Publication bias was estimated by funnel plot, Egger's test, and fail-safe numbers.

**Results:**

35 studies with 4,401 participants with diabetes were included in the meta-analysis. Glycated hemoglobin A levels were elevated in patients with Helicobacter pylori infection compared with patients without Helicobacter pylori infection (WMD = 0.50, 95% CI: 0.28-0.72, *p* < 0.001). In subgroup analysis by the subtype of diabetes, there was a correlation between Helicobacter pylori infection and elevated glycated hemoglobin A in type 1 diabetes (*I*^2^ = 74%, *p* < 0.001, WMD = 0.46, 95% CI: 0.12-0.80), and in type 2 diabetes (*I*^2^ = 90%, *p* < 0.001, WMD = 0.59, 95% CI: 0.28-0.90, *p* < 0.001). In subgroup analysis by the study design, there was a correlation in cross-sectional study (*I*^2^ = 89%, *p* < 0.001, WMD = 0.42, 95% CI: 0.16-0.69, *p* ≤ 0.003) and in case-control study (*I*^2^ = 83%, *p* < 0.001, WMD = 0.39, 95% CI: 0.14-0.64, *p* ≤ 0.003). By different methods for detecting Helicobacter pylori, there was a correlation in the biopsy group (*I*^2^ = 83%, *p* < 0.001, WMD = 0.6, 95% CI: 0.11-1.09, *p* ≤ 0.03) and in other groups of test methods (*I*^2^ = 87%, *p* < 0.001, WMD = 0.37, 95% CI: 0.17-0.56, *p* < 0.001). Sensitivity analysis showed that our results were reliable, and no evidence of substantial publication bias was detected.

**Conclusion:**

The meta-analysis might indicate a correlation between Helicobacter pylori infection and glycated hemoglobin A levels in diabetes.

## 1. Introduction

In 2007, the International Federation of Clinical Chemistry (IFCC) clearly defined glycated hemoglobin A (HbA1c) as a stable adduct formed by glucose and the free amino group of the hemoglobin *β* chain-N-terminal proline [1]. In 2011, World Health Organization officially recommended HbA1c ≥ 6.5% as a diagnostic cutoff point for diabetes [2]. The American Diabetes Association (ADA) recommends that HbA1c should be measured in patients with newly developed diabetes, and it plays an important role in the monitoring of diabetes as an evaluation index to judge the effect of blood glucose control. Helicobacter pylori infection is now considered the most important cause of gastritis and peptic ulcer in humans. And studies have reported on the potential links between H. pylori infection and a variety of extra-gastroduodenal manifestations ischemic as heart disease, liver diseases, skin diseases, blood disorders, neurologic disorders, and others [3]. Christie et al. found serological evidence of H. pylori infection which was associated with an increased rate of incident diabetes in a Latino elderly cohort [4]. However, studies on the relationship between H. pylori infection and HbA1C in diabetic patients are inconsistent and sometimes contradictory. The finding of Bazmamoun et al. showed that there was no correlation between Helicobacter pylori infection and HbA1c levels [5]. Studies by Akın et al. found that HbA1c levels in Helicobacter pylori-positive patients were significantly higher than those in Helicobacter pylori-negative patients [6]. Due to these discrepancies, we performed a meta-analysis investigating the relationship between H. pylori infection and glycated hemoglobin A in patients with diabetes.

## 2. Methods

### 2.1. Literature Search Strategy

“Helicobacter pylori” and “glycated hemoglobin A” were jointly searched in PubMed database, the Embase database, and China National Knowledge Infrastructure (CNKI) as keywords for all relevant literature published before April 2019. Moreover, we also reviewed the reference not captured by our database search.

### 2.2. Inclusion Criteria

The inclusion criteria were as follows: (1) observational studies; (2) studies are related to the relationship between H. pylori and diabetes, including case groups and control groups, and providing the exact sample size, the number of patients with H. pylori infection, and the mean and standard deviation of the level of HbA1c; (3) the diagnosis of diabetes was in agreement with international guidelines [7]; (4) H. pylori infection is judged by at least one diagnostic method; and (5) the studies are not directly related but with the abovementioned requirements.

### 2.3. Exclusion Criteria

The exclusion criteria were as follows: (1) case report and observational studies without control groups; (2) studies in which the data of the level of HbA1c were not available for either diabetes group or control group; (3) subset of a published article by the same authors or repeated published literature; (4) studies limited to animal; and (5) the data of literature are incomplete with little information, and the extraction of original data is not enough to calculate the statistics of this study.

### 2.4. Study Selection

Two researchers independently screened the literature, extracted the data, and cross-checked. If the results were inconsistent, those would be discussed together or judged by a third senior researcher. This study used pre-established data extraction forms to extract data from the literature that will eventually be included in the meta-analysis. The excerpts included the first author, the year of publication, the study area, the diagnostic criteria for H. pylori infection, the sample size of the case and control groups, and the mean and standard deviation of HbA1c.

### 2.5. Statistical Analysis

The data and the database were organized and checked carefully according to the requirements of the meta-analysis. RevMan 5.3 was used for statistical analysis, and weighted mean difference (WMD) with 95% CI was used for quantitative analysis of measurement data. *I*^2^ was used to quantitatively test the heterogeneity among different studies. If *I*^2^ ≤ 50%, the heterogeneity had no statistical significance, and fixed effects model was used to analyze it. On the contrary, if *I*^2^ > 50% the heterogeneity had statistical significance, and random effects model was used to analyze. Moreover, subgroup analysis was carried out to explore the sources of heterogeneity according to the factors that might produce heterogeneity. To ensure the stability of the results of the meta-analysis, the sensitivity analysis (after the included studies removed one by one, the combined analysis was performed again, and the significant difference between the effect values before and after the combination was compared) was performed. The funnel plot, Egger's test, and fail-safe number were used to quantitatively evaluate the publication bias. *p* < 0.05 was considered statistically significant, suggesting that publication bias is not excluded.

## 3. Results

### 3.1. Study Selection and Characteristics

**Figure 1:**
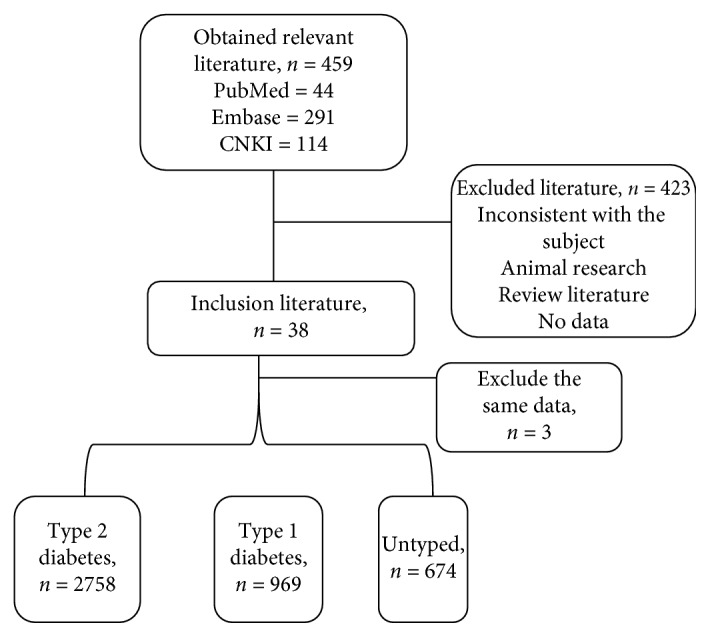
Flow chart of study selection.

A total of 459 articles were initially searched by terms, and 35 studies eventually met the predetermined inclusion and exclusion criteria (Figure 1). The relevant literature was published from 2000 to 2018 (Table 1). A total of 4,401 diabetic patients were included in the meta-analysis, including 1176 patients with type 1 diabetes, 2877 patients with type 2 diabetes, and 348 patients who were not typed. The included literature included 20 case-control studies involving 1970 patients with diabetes and 15 cross-sectional studies involving 2,431 people with diabetes.

### 3.2. Results of Meta-Analysis

**Figure 2:**
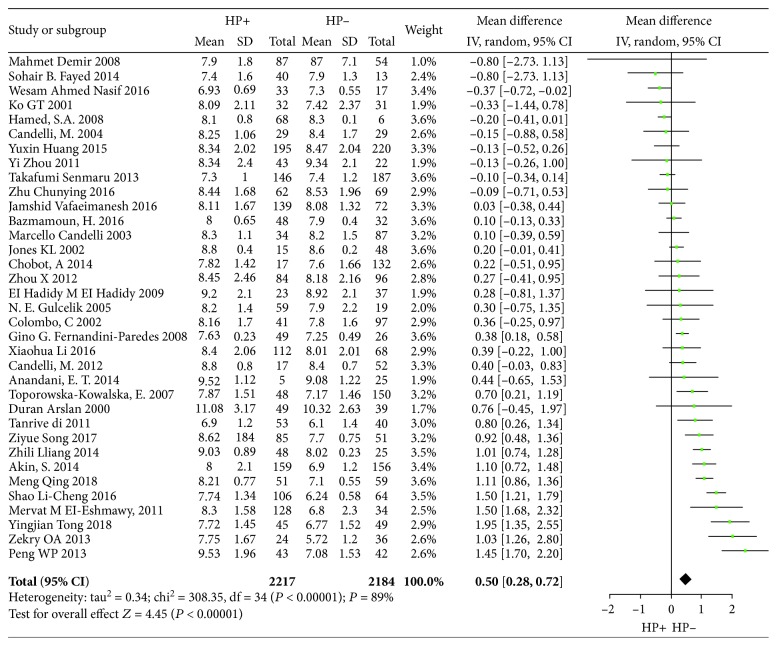
Forest plot of Helicobacter pylori infection and glycated hemoglobin level analysis.

RevMan 5.3 was used to test the heterogeneity, *I*^2^ = 89%, *p* < 0.001, so the random effects model was used to conduct a combined analysis. 35 studies (WMD = 0.50, 95% CI: 0.28-0.72, *p* < 0.001) showed that there was a significant difference in the level of HbA1c between patients infected with H. pylori and that of noninfected patients. H. pylori infection was correlated with the increased level of HbA1c (Figure 2).

### 3.3. Subgroup Analysis

**Figure 3:**
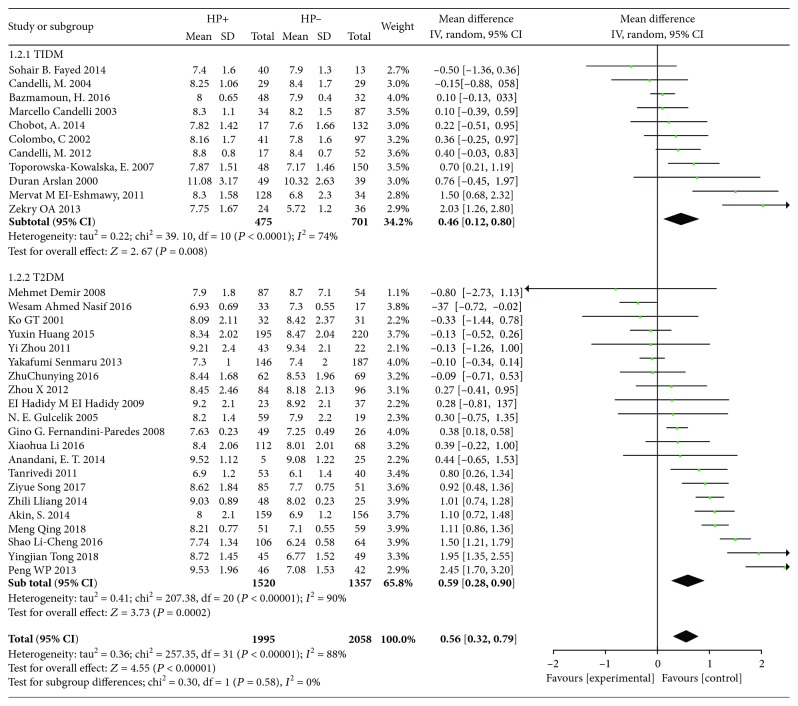
Forest plot of Helicobacter pylori infection and glycated hemoglobin level: subgroup analysis grouped by disease classification.

**Figure 4:**
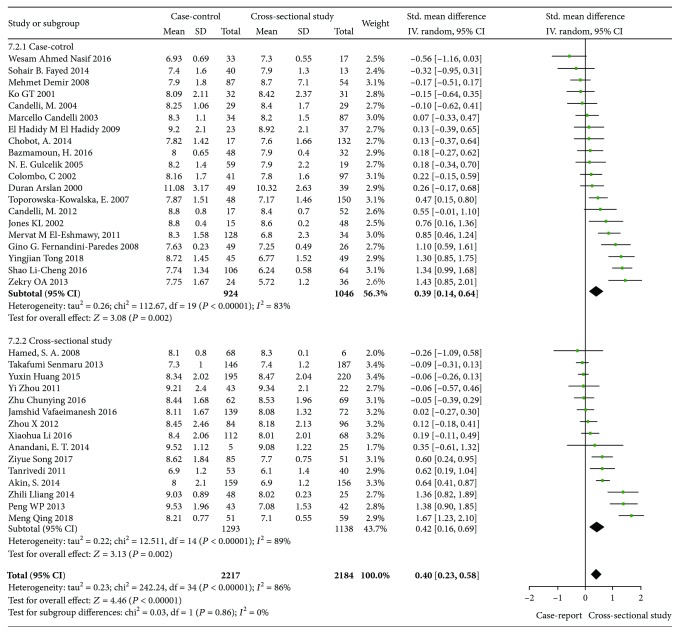
Forest plot of Helicobacter pylori infection and glycated hemoglobin level: subgroup analysis forest map, grouped by study type.

**Figure 5:**
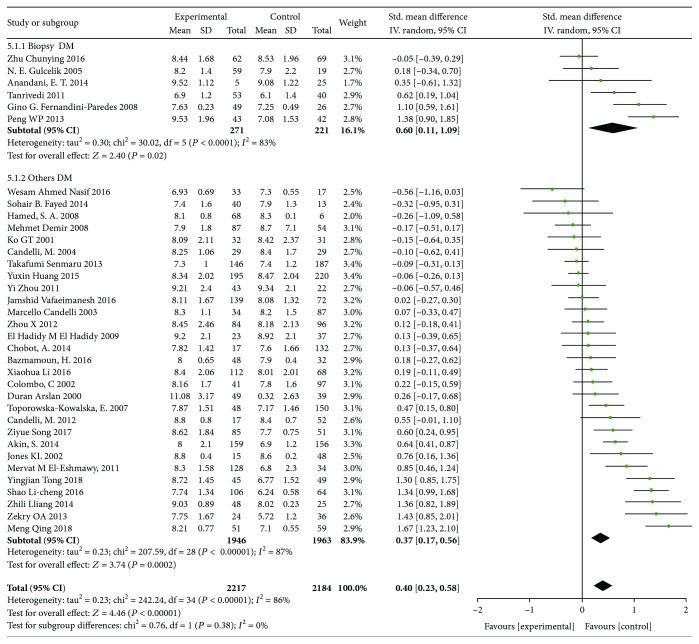
Forest plot of Helicobacter pylori infection and glycated hemoglobin level: subgroup analysis, grouped by detection method.

In order to further increase the reliability of the study, the subtypes of diabetes mellitus, the design of the studies and different detection methods of H. pylori were analyzed, which were divided into three subgroups: type 1 diabetes mellitus and type 2 diabetes mellitus; cross-sectional studies and case-control studies; and biopsy and other detection methods. The results of the analysis are shown in Figures 3–5. (1) In subgroup analysis by the type of diabetes, it is indicated that there is a significant correlation between H. pylori infection and increased HbA1c in type 1 diabetic patients (*I*^2^ = 74%, *p* < 0.001, WMD = 0.46, 95% CI: 0.12-0.80) and in type 2 diabetes (*I*^2^ = 90%, *p* < 0.001, WMD = 0.59, 95% CI: 0.28-0.90, *p* < 0.001). (2) In subgroup analysis by design of studies, 15 of them were cross-sectional studies, of which 2217 were infected with H. pylori and 2184 were uninfected. 20 studies were case-control studies, of which 524 patients with H. pylori infection and 1046 patients without. A total of 35 studies were analyzed by meta-analysis. It is showed that H. pylori infection was associated with HbA1c in cross-sectional study (*I*^2^ = 89%, *p* < 0.001, WMD = 0.42, 95% CI: 0.16-0.69, *p* ≤ 0.003) and in case-control study (*I*^2^ = 83%, *p* < 0.001, WMD = 0.39, 95% CI: -0.14-0.64, *p* ≤ 0.003). (3) Gastric biopsy group and other detection methods group: biopsy was used in 6 studies and other detection methods were used in 29 studies, including serological detection and breath test. There was statistical significance in the biopsy group (*I*^2^ = 83%, *p* < 0.001, WMD = 0.6, 95% CI: 0.11-1.09, *p* ≤ 0.03) and in other groups of test methods (*I*^2^ = 87%, *p* < 0.001, WMD = 0.37, 95% CI: 0.17-0.56, *p* < 0.001). It shows that H. pylori infection is correlated with the level of HbA1c whether in biopsy or other methods.

## 4. Publication Bias

**Figure 6:**
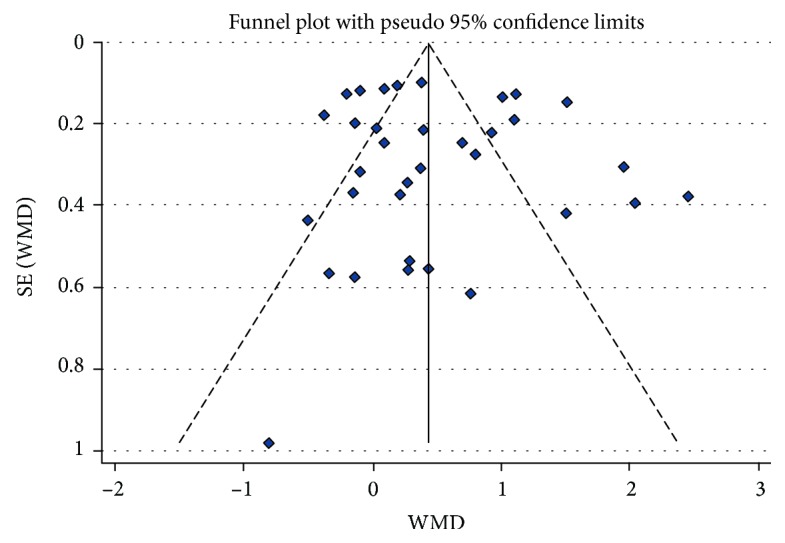
Publication bias test: funnel plot.

Egger's test (*p* > 0.05) showed no significant publication bias. Funnel plot is basically symmetrical and has no publication bias (Figure 6). Fail-safe numbers, indicating the publication bias, are reported in Table 2. The fail-safe numbers were all relatively large in the meta-analysis, suggesting that the results were reliable.

## 5. Discussion

The quantitative data of this meta-analysis showed that the level of HbA1c in the H. pylori-infected group was significantly higher than that in the H. pylori-negative group, indicating that H. pylori infection and HbA1c were correlated in diabetes. Subgroup analysis revealed that (1) Helicobacter pylori infection was correlated with the level of HbA1c in type 1 diabetes mellitus and type 2 diabetes mellitus. (2) According to the type of studies, they were divided into two subgroups: case-control study and cross-sectional study. There was statistical significance in cross-sectional studies and case-control studies. (3) According to the diagnostic criteria of H. pylori infection, the H. pylori infection was correlated with the level of HbA1c in the gastric biopsy group. Similarly, the differences in other test groups were also statistically significant.

The results of this study are not completely consistent with the results of a meta-analysis of Dai et al. [41] in 2015. Their 11 studies showed that HbA1c of type 1 diabetic patients with H. pylori infection was significantly higher than that of type 1 diabetic patients without H. pylori infection (WMD = 0.35, 95% CI: 0.05-0.64; *p* ≤ 0.03). However, there was no significant difference in the levels of HbA1c between type 2 diabetes mellitus with and without H. pylori infection (WMD = 0.51, 95% CI: -0.63-1.65; *p* ≤ 0.38). According to the results of increasing sample size, both type 1 diabetes mellitus and type 2 diabetes mellitus patients infected with H. pylori have high levels of HbA1c and poor control of glycemic indices.

It is estimated that about 4.4 billion people worldwide were infected with H. pylori in 2015 [42], and it is estimated that diabetes will reach 552 million by 2020 [43]. Kato et al. have shown that H. pylori infection is associated with an increased risk of diabetes mellitus [44]. Refaeli et al. showed that the prevalence of metabolic syndrome in H. pylori-infected patients was higher than that in uninfected patients [45]. More and more data indicated that inflammation may play a role in the pathogenesis of type 2 diabetes, and the pathogenesis of type 2 diabetes can be regarded as an autoinflammatory disease [46]. Simultaneously, the inflammatory response caused by H. pylori has also been confirmed by researches [47, 48]. At the same time, studies have shown that eradication of H. pylori can improve glucose homeostasis in type 2 diabetes mellitus by reducing proinflammatory factors [49]. In addition, studies have shown that H. pylori can promote insulin resistance by inducing chronic inflammation and affecting insulin regulation of gastrointestinal hormones [50]. Gastritis caused by H. pylori may affect the secretion of gastric-related hormones, such as leptin and growth hormone-releasing hormone, as well as gastrin and somatostatin, which may affect the susceptibility to diabetes [51]. Studies have also described a positive correlation between H. pylori infection and impaired insulin secretion [52]. The results of Zhou et al. showed that H. pylori infection induced hepatic insulin resistance through c-Jun/miR-203/SOCS3 signaling pathway and provided possible implications for insulin resistance [53]. Although the mechanism of the association between H. pylori infection and diabetes is still unclear, more and more studies have shown that there is a potential link between them. Because of the large number of patients with the two diseases, if the causal relationship between them becomes clear, the impact will be great.

Meta-analysis is a secondary literature analysis based on previous research evidence. Therefore, there are limitations and biases in the analysis. Case-control studies are inevitably affected by selective bias. The results are not as reliable as prospective studies, and there are some limitations. Moreover, the results of this study are not grouped by population, region, and race. The limitation of this meta-analysis is that it does not consider other characteristics that may affect blood sugar control besides H. pylori infection, such as treatment status, age, gender, obesity index, or smoking status.

In conclusion, the results of this meta-analysis indicate that Helicobacter pylori infection is associated with increased glycosylated hemoglobin A, with a large sample size and a certain degree of confidence. Although some biases affect the accuracy of the results, it is still possible to provide new reference and guidance for eradication of H. pylori as a secondary prevention or treatment of diabetes. Proper screening for H. pylori infection and regular monitoring of blood glucose and HbA1c may be effective for early detection of blood glucose disorders and prevention of type 2 diabetes. Further research, especially longitudinal studies, is necessary to validate current results.

## 6. Conclusion

This study conducted a meta-analysis of existing literature and concluded that H. pylori infection may increase the level of glycosylated hemoglobin A in diabetic patients, and the same conclusions were obtained in both type 1 diabetes and type 2 diabetes. The sample size included in this paper is large and has credibility, which can guide clinical work to a certain extent. In clinical practice, individualized prevention and treatment need to be closely combined with the actual situation of the patient.

## Figures and Tables

**Table 1:** 

Authors	Year	Country	Type	HP+		HP−	
				Mean ± SD (%)	*n*	Mean ± SD (%)	*n*
Chobot et al. [8]	2014	Poland	T1DM	7.8 ± 1.42	17	7.60 ± 1.66	132
Fernandini-Paredes et al. [9]	2008	Peru	T2DM	7.6 ± 0.23	49	7.25 ± 0.49	26
Hamed et al. [10]	2008	Egypt	T1DM/T2DM	8.1 ± 0.8	68	8.3 ± 0.1	6
Candelli et al. [11]	2004	Rome	T1DM	8.2 ± 1.06	29	8.4 ± 1.7	29
Yingjian [12]	2018	China	T2DM	8.7 ± 1.45	45	6.77 ± 1.52	49
Qing et al. [13]	2018	China	T2DM	8.2 ± 0.77	51	7.10 ± 0.55	59
Ziyue et al. [14]	2017	China	T2DM	8.6 ± 1.84	85	7.70 ± 0.75	51
Chunying et al. [15]	2016	China	T2DM	8.4 ± 1.68	62	8.53 ± 1.96	69
Licheng et al. [16]	2016	China	T2DM	7.7 ± 1.34	106	6.24 ± 0.58	64
Li [17]	2016	China	T2DM	8.4 ± 2.06	112	8.01 ± 2.01	68
Zhili et al. [18]	2014	China	T2DM	9.0 ± 0.89	48	8.02 ± 0.23	25
Yi et al. [19]	2011	China	T2DM	9.21 ± 2.4	43	9.34 ± 2.1	22
Candelli et al. [20]	2003	Rome	T1DM	8.3 ± 1.1	34	8.2 ± 1.5	87
Fayed et al. [21]	2014	Egypt	T1DM	7.4 ± 1.6	40	7.9 ± 1.3	13
Vafaeimanesh et al. [22]	2016	Iran	T1DM/T2DM	8.1 ± 1.67	139	8.08 ± 1.32	72
Nasif et al. [23]	2016	Saudi Arabia	T2DM	6.9 ± 0.69	33	7.30 ± 0.55	17
Anandani et al. [24]	2014	Indonesia	T2DM	9.52 + 1.12	5	9.08 + 1.22	25
Toporowska-Kowalska et al. [25]	2007	Poland	T1DM	7.87 ± 1.51	48	7.17 ± 1.46	150
Bazmamoun et al. [6]	2016	Iran	T1DM	8 ± 0.65	48	7.90 ± 0.40	32
Huang et al. [26]	2015	China	T2DM	8.34 ± 2.02	195	8.47 ± 2.04	220
Demir et al. [27]	2008	Turkey	T2DM	7.9 ± 1.8	87	8.7 ± 7.1	54
Arslan et al. [28]	2000	Turkey	T1DM	11.08 ± 3.17	49	10.32 ± 2.63	39
Colombo et al. [29]	2002	Italy	T1DM	8.16 ± 1.7	41	7.8 ± 1.6	97
Gulcelik et al. [30]	2005	Turkey	T2DM	8.2 ± 1.4	59	7.9 ± 2.2	19
Zekry et al. [31]	2013	Egypt	T1DM	7.75 ± 1.67	24	5.72 ± 1.2	36
Ko et al. [32]	2001	China	T2DM	8.09 ± 2.11	32	8.42 ± 2.37	31
Jones et al. [33]	2002	Australia	DM	8.8 ± 0.4	15	8.6 ± 0.2	48
Candelli et al. [34]	2012	Italy	T1DM	8.8 ± 0.8	17	8.4 ± 0.7	52
Zhou et al. [35]	2012	China	T2DM	8.45 ± 2.46	84	8.18 ± 2.13	96
El-Eshmawy et al. [36]	2011	Egypt	T1DM	8.3 ± 1.58	128	6.8 ± 2.3	34
Senmaru et al. [37]	2013	Japan	T2DM	7.3 ± 1.0	146	7.4 ± 1.2	187
Tanrivedi [38]	2011	Turkey	T2DM	6.9 ± 1.2	53	6.1 ± 1.4	40
Peng et al. [39]	2013	China	T2DM	9.53 ± 1.96	43	7.08 ± 1.53	42
Akın. et al. [6]	2014	Turkey	T2DM	8.0 ± 2.1	159	6.9 ± 1.2	156
El Hadidy et al. [40]	2009	Saudi Arabia	T2DM	9.2 ± 2.1	23	8.92 ± 2.1	37

**Table 2:** Fail-safe numbers of all groups for the studies.

	Number of studies	Fail-safe number	
		*α* = 0.05	*α* = 0.01
All diabetic patients	35	1799.584	873.894
Subgroup of type 1 diabetes	11	101.251	44.612
Subgroup of type 2 diabetes	21	936.340	453.288
Subgroup of case-control	20	491.393	233.356
Subgroup of cross-sectional study	15	393.769	187.513
Subgroup of biopsy	6	78.977	36.010
Subgroup of other methods	29	1100.883	530.770
